# Barriers to Exercise in Younger and Older Non-Exercising Adult Women: A Cross Sectional Study in London, United Kingdom

**DOI:** 10.3390/ijerph6041443

**Published:** 2009-04-15

**Authors:** Walid El Ansari, Geoff Lovell

**Affiliations:** 1 Faculty of Sport, Health & Social Care, University of Gloucestershire, Gloucester, UK; 2 School of Social Sciences, Faculty of Arts and Social Sciences, University of the Sunshine Coast, Queensland, Australia; E-Mail: glovell@usc.edu.au

**Keywords:** Physical activity, exercise barriers, women, child care, obesity

## Abstract

A survey of 100 women in the south of London, United Kingdom (UK) compared exercise barrier intensities between non-exercising younger (20–27 years) and older (28–35 years) adult women; and examined childcare duties as perceived barriers to exercise. Perceived barriers to exercise were examined using an Exercise Benefits/Barriers Scale (EBBS) comprising four subscales (exercise milieu; time expenditure; physical exertion; family discouragement). Participants’ number of children was also noted. Non-exercising older women reported significantly higher total exercise barriers, as well as across three barrier subscales: exercise milieu, time expenditure, and family discouragement. For both age groups, significant correlation existed between number of children and women’s total exercise barrier scores. Number of children explained ≈25% and ≈30% of the variance of younger and older women’s total barrier scores respectively. For both women groups, the strongest correlation between exercise barrier and number of children was for the time expenditure subscale. Broad grouping of 20–35 year old non-exercising women does not reflect a homogenous sample. Age categories employing narrower age brackets are recommended. Issues surrounding family responsibilities e.g. childcare duties may be shared between these groups and require further research and policy attention.

## 1. Introduction

Obesity and physical activity (PA) levels are both important concerns for young women. Obesity in women is a major global health threat [1], but also a reversible predisposing factor for diabetes, coronary artery disease, hypertension, osteoarthritis, colon cancer and premature mortality [2–4]. Obesity in young adult women in the UK is increasing; in 2002, 22.8% of adult women were clinically obese [5], a threefold increase since 1980. Today in England alone, nearly a quarter of women are now considered obese [6]. Reports suggest that by 2010 a third of all UK adults will be obese [7], matching current levels in the USA, where being overweight at age 25 in African-American and white women was associated with early retirement for health reasons [8]. Furthermore the rate of obesity in women is significantly higher than men, and obesity in females increases with increasing age [2]. This obesity boom, once an urban occurrence, has now quickly spread to rural populations [9].

Women’s PA levels are also concerning; at all ages women are less physically active than males [10]. The risk of obesity is inversely related to the level of regular exercise, where women exercising 3–4 hours per week were ≈60% less likely to be obese compared to those who do not exercise [2]. Indeed, a review reported that PA at levels that are attainable by ‘ordinary’ people is preventive for cardiovascular conditions [11]. Despite the paybacks of regular PA as a stroke and cardiovascular disease prevention strategy, middle-aged and older Latin-American women persist to be physically inactive and obese [12].

The acknowledgment of the role of PA in improving health has led recent policies to focus explicitly at PA promotion to increase the PA levels [13,14]. In the UK, this led to the development of community PA programmes, e.g. physical activity referral schemes (also termed exercise referral schemes) [15]. In such schemes, health professionals refer people to undertake 10–12 weeks of PA that is supervised by qualified exercise professionals [16,17]. Despite such initiatives, participants often removed themselves from the schemes (no contact); chose not to proceed with the referral; or were assigned to a leisure provider but failed to attend or to complete the programme [18]. Further, the odds of participants taking up referral increased with age, where those aged 50–69 years were more likely to uptake the referral compared with the younger age groups (≤ 29 years and 30–39 years) [18]. Such patterns suggest barriers to participation in PA programmes for the younger groups. Given that recent guidance on PA interventions recommended stopping the use of PA referral schemes other than for controlled research [19], and national PA recommendations have made small progress in diminishing the proportion of sedentary adults [20], this stipulates that progress would be of even smaller magnitude in non-exercising adult women.

But why do people not participate in PA and exercise? The perceived benefits and barriers to exercise have been suggested as significant mediators for PA behaviour change [21]. Vaughn [12] examined the factors that influenced women’s participation in PA and found that the facilitators and barriers were identified as primary categories which were further classed into intrinsic or extrinsic factors. Furthermore, individuals who perceive more exercise benefits and fewer exercise barriers are typically more active than those who report high perceived barriers and low perceived benefits [21].

The importance of minimizing the barriers to exercise echoes with recent policies aimed at enhancing peoples’ PA levels [13,14], and concurs with a review (≈50 studies) of health behaviour change where perceived barriers were the single most powerful predictors of health behaviour [22]. However, such barriers to exercise have not been examined in detail [23], and studies tended to exclude women, even though women are typically less active than men [24]. The long-term success of strategies to increase adult women’s PA has been insufficient [25], hence understanding the benefits/ barriers [4] could overcome the limited evidence base that constrains PA promotion efforts and primary prevention strategies for midlife women [23,26].

Several hypotheses have been postulated to explain women’s lower PA levels, e.g. social factors; care-giving duties; and access to exercise opportunities. However the limited understanding of barriers to PA for women [27]; and the different barriers to exercise for women across the lifespan [24] both remain under-researched. For instance, for adult women, few studies examined how perceived barriers to exercise may differ between younger (20–27 years old) and older (28–35 years old) adult females [28]. Previous research observed barriers reported by either younger (15–34 years) or older females (35–54 years of age). However, developmental milestones or the circumstances of women at different stages of their lives impacts on both their interests in exercise and ability to be physically active [27]. The paucity of information related to exercise barrier intensity for these younger and old older adult women creates problems for PA promotion efforts and policy makers aiming to the increase PA levels in these key target groups.

### 1.1. Aims of the Study

This research aimed to assess the barriers to PA and their relationships to family responsibilities in two groups of adult women. The specific objectives were: (1) to compare the exercise barrier intensity between non-exercising *younger* adult women (20–27 years of age) and non-exercising *older* adult women (28–35 years of age) as measured by the Exercise Benefits/Barriers Scale (EBBS) [29]; and (2) to further the body of knowledge regarding how specific family responsibilities contexts such as childcare duties are associated with perceived exercise barrier scores in these two adult women groups.

## 2. Methods

### 2.1. Sample and Participants

Following ethical approval by the Kingston University Ethics Committee, a shopping centre in the south west of London, UK was randomly selected, and 100 women at the shopping centre were then randomly selected. Women were eligible to participate in this study if they reported to be non-exercising, free from disease, and aged between 20–35 years. ‘Non-exercising’ was defined as an individual not meeting the American College of Sports medicine recommendation of least 30 minutes of moderate exercise carried out over the course of most days of the week [30].

Data collection was undertaken in the month before Christmas 2007, on three separate occasions comprising a Saturday afternoon, a Wednesday afternoon, and a Thursday evening (for late night shoppers). The study approached every 10^th^ women at the shopping centre that on first look appeared to fall within the age range specific to the study. Each potential participant that was approached was informed that as part of a research projected associated with the University, women aged 20–35 who did not partake in 30 minutes of moderate intensity exercise over most days of the week were being invited to complete a short questionnaire. Potential respondents were informed about the aims of the survey, and that participation entails answering some short questions about their thoughts towards barriers to exercise, as well as some demographic/background questions. Participants were asked if they fitted the inclusion criteria, if so they were then informed that participation in the study was voluntary, all information was confidential, no record of respondents’ names would be made, and that participants were free to withdraw at any point that they wished to do so. If in agreement the participant then completed the survey. Recruitment continued until two groups were obtained: one consisting of 50 younger adult women (aged 20–27 years); and the second comprising 50 older adult women (aged 28–35 years). Roughly, 80% of the women approached agreed to participate in the study.

### 2.2. Instrument

The only measure of PA level was whether participants achieved or did not achieve the 30 minutes of moderate exercise over most days of the week. Perceived barrier intensities to exercise were assessed by the barriers component of the EBBS questionnaire [29]. The EBBS has two components: benefits and barriers. The barrier component comprised 14 barrier items categorised into four subscales: exercise milieu; time expenditure; physical exertion; and family discouragement, with high internal consistency (0.87) and test re-test reliability (0.77) [29]. The specific items comprising each subscale are depicted in Box (1).

The barrier aspect of the EBBS can be safely used in isolation (i.e. without the benefits component) [29]. Given that barriers are more predictive of exercise behaviour than benefits [7], for the current investigation only the barrier component of the EBBS was utilized. Each of the 14 barrier items were scored on a 4-response Likert format (1 = strongly disagree; 2 = disagree; 3 = agree; and, 4 = strongly agree). Scores for total barrier and each of the barrier sub-scales were adjusted to the same 1 to 4 Likert scale to allow direct comparison between barrier sub-scales. Hence a high score represented more perception of barrier and vice versa.

### 2.3. Statistical Analyses

A single MANOVA assessed differences between younger and older non-exercising adult women’s barrier intensities, with the total barrier score and the four subscales constituting the multivariate aspect. Justified by a significant MANOVA group main effect, subsequent univariate tests were conducted upon the total barrier as well as the individual subscales (exercise milieu, time expenditure, physical exertion, and family discouragement barrier scores) to provide a detailed understanding of the differences between the groups. Separate Pearson bivariate correlations were undertaken between total EBBS barrier scores and participant’s number of children for each group in order to assess the association of family responsibilities (e.g. childcare duties) with perceived exercise barrier intensity scores in both younger and older non-exercising adult women groups. The analysis was performed with SPSS^®^ for Windows version 12. For all tests, significance level was set at p < 0.05.

**Box 1. Perceived exercise barriers: eleven items.**

**Exercise Milieu subscale (6 items)**

1. Places for me to exercise are too far away
2. I am too embarrassed to exercise
3. It costs too much money to exercise
4. Exercise facilities do not have convenient schedules for me
5. I think people in exercise clothes look funny
6. There are too few places for me to exercise

**Time Expenditure subscale (3 items)**

7. Exercise takes too much time from family relationships
8. Exercise takes too much time from my family responsibilities
9. Exercising takes too much of my time

**Physical Exertion subscale (3 items)**

10. Exercise tires me
11. I am fatigued by exercise
12. Exercise is hard work for me

**Family Discouragement subscale (2 items)**

13. My spouse (or significant other) does not encourage exercising
14. My family members do not encourage me to exercise

All items scored on 4-response Likert scales.

## 3. Results

### 3.1. Demographic Characteristics of the Sample

Participants (N = 100) were women randomly present in a shopping centre (mall) in the south west of London, UK. Response rate for those who were eligible to participate in the survey (i.e. non-exercising, free from disease, and aged 20–35 years) was ≈80%. Table 1 shows participants’ age, number of children, educational attainment, and socio-economic characteristics.

Given the significant MANOVA group main effect [F (4,95) = 2.76, *p* < 0.05], subsequent univariate analysis was undertaken and showed that the older women reported significantly higher total barrier scores [*F* (1,98) = 6.42, *p* < 0.05]. Particularly, older women reported significantly higher scores for the exercise milieu [*F* (1,98)= 4.48, *p* < 0.05], time expenditure [*F* (1,98) = 8.39, *p* < 0.01], and family discouragement [*F* (1,98) = 4.66, *p* < 0.05] barrier subscales. Older women also rated higher scores for the physical exertion barrier, but the difference was not significant. Table (2) depicts the perceived exercise barrier scores for both groups of women.

Pearson’s correlations showed that participant’s number of children was associated with the women’s perceived exercise barrier intensity scores. For the total barrier score, there were significant correlations in both the younger (*r* = 0.50, *p* < 0.001) and older (*r* = 0.56, *p* < 0.001) non-exercising women. These strong relationships showed that participant’s number of children explained 25% (*r*^2^) of the variance of the younger women’s total barrier scores, and > 30% of the older women’s total barrier scores. Of the four barrier subscales, only family discouragement was not significantly correlated with women’s number of children (*p* = 0.052 for younger women; *p* = 0.128 for older women). The strongest correlations between the exercise barrier intensity score and number of children for both the younger and older women were for the time expenditure scale (*r* = 0.65, *p* < 0.001 and *r* = 0.59, *p* < 0.001 respectively).

## 4. Discussion

On current trends, by 2050 about 50% of females in England could be obese [31]. Similarly, the decreasing PA levels are well documented in women [32]. Within this context, the objectives of this study were to compare exercise barrier intensities between non-exercising younger and older adult women, and to further the understanding of how family responsibilities (e.g. childcare duties) might be associated with the perceived exercise barrier scores in these adult groups.

In relation to the first objective, older female adults perceived significantly greater barriers to exercise than their younger counterparts. This finding suggests that the broad grouping of 20–35 year old non-exercising women as undertaken in many studies (e.g. [33]) does not reflect a homogenous sample, as the lifestyle challenges faced by women at either end of this age bracket are likely to be different, probably due to career and family responsibilities (e.g. childcare commitments) [25]. Our findings support the calls that research targeting gender and different life stages is necessary [34,35]. The use of wide age brackets could conceal important existing differences in PA levels, underestimate the intensity of perceptions to exercise barriers, or contribute to erroneous findings. In order to understand exercise barriers in adult women so that PA programmes are better targeted, more sensitive age-grouping of participants is mandatory, employing narrower age brackets. This proposition is further supported by findings that midlife women exhibited a higher risk of being inactive compared to younger women [36,37]. This has implications for future research as well as efforts and policy developments to increase PA for women along the continuum of ages 20–35 years.

Besides that older women reported significantly higher total barrier intensities, they also rated significantly higher three of the four individual barrier subscales that were examined: exercise milieu (exercise situation); time expenditure; and family discouragement. These differences may reflect the different circumstances confronting women at different stages of their lives, and how these factors impact on both women’s interests in exercise and their ability to be physically active [22]. Such lifespan changes include the individual’s attitude to PA and exercise, diverse lifestyle features, differing priorities, dissimilar amount of free-time, pending/ pressing career commitments, increasing family responsibilities, and placing the family’s financial security and wellbeing as a first concern over other functions including PA. Our findings agree with others [38] who showed that as regards women’s exercise, parenthood affected exercise participation most and that its effect was dependent on age. However, a point to note in our study was that there was no significant difference between the age groups for the physical exertion barrier. This suggested that it was not the physical effort *perse* associated with PA that forms a differential barrier to exercise between these groups. Rather the difference seems to stem from, in terms of perceived barrier to exercise, as a result of other factors such as time available to be physically active, family responsibilities or discouraging family environment/ features. Indeed women’s social roles e.g. parenthood and employment status and how they influence PA have received some attention [38].

The correlation analysis demonstrated significant relationships for the younger adult women, where their number of children accounted for ≈25% of the variance in their total EBBS barrier score, while for the older women their number of children accounted for ≈30% of the variance in their total barriers to exercise. This is in agreement with Albright *et al.* [39] who highlighted the potentially ‘negative’ impact motherhood may have upon PA, with only 35% of women who were active before childbirth, being active after childbirth. This is in the face that women display a 20% chance of having a stroke during their lifetime, and younger women in childbearing years, pregnancy, and use of oral contraceptives have possible risks [40]. Hence improving stroke prevention in women would include exercising regularly and maintaining a healthy BMI to mitigate the risks of physical inactivity and obesity [41]. Indeed PA is an important action to prevent cardiovascular conditions, women’s foremost cause of death [42,43], and also prevents other expensive conditions that appear as women age (e.g. osteoporosis, cognitive decline, depression, and breast cancer) [44,45]. Unsurprisingly, UK policies increasingly aim at sets of programmes to ensure that there is a clear legacy of increased PA and the building of PA into people’s lives [6].

Considering that the older women group in the current investigation had a higher mean number of children, this may contribute to explaining their significantly higher barriers in relation to exercise milieu, time expenditure, and family discouragement scores. This supports others [46–48], where women commonly reported that family priorities, care giving duties, and lack of time were barriers to participation in PA. Even for employed mothers who are at work, having children and the lack of energy were significant barriers to women’s participation in workplace exercises [49].

An inspection of the individual items of the EBBS suggested logical links between the number of children a women has and her EBBS barriers subscale scores. E.g., for older adult women with more children, the exercise milieu for opportunities to be physically active was seen as more demanding. These women may perceive less favourably the operating times of fitness studios or the distances and travel time to exercise venues. They might feel that actual out-of-pocket financial costs of fitness venues could be well spent on family commitments or other requirements, or alternatively, that such costs might be seen as less conducive (high barriers) for a healthier lifestyle (e.g., “Places for me to exercise are too far away”; “Exercise facilities do not have convenient schedules for me”; “It costs too much to exercise”). Overcoming these barriers requires higher levels of PA motivation, behavioural regulation, participation [50], and high commitment to sustain a momentum towards goals that would forecast exercise participation [51]. Such goals that are chosen would also need to promote women’s ‘self-worth’ values so that they do not themselves become additional barriers resulting in a vicious circle: i.e. should not induce high psychological costs [52] or result in a decreased motivational state [53].

Similarly from a time expenditure point of view, older adult women are likely to have more children, with corresponding more demanding/ continuous family interactions (e.g., “Exercise takes too much time from family relationships”) and may also have wider domestic duties, school runs, or familial/ household errands etc. Such factors may partly explain why people find it tricky to adhere to and incorporate exercise as a habitual part of their lives [54]. If transport, time management, finances, and family commitments become increasingly pressured by motherhood, it becomes plausible that the number of children a mother has could be directly related to her EBBS barrier scores. Women’s care-giving responsibilities and roles, coupled with women’s significantly lower exercise rate [37], might contribute to explain women’s different and more intense barriers to habitual PA than men. This suggests that women who are blending several roles, tasks and responsibilities would benefit from self-regulation tools: e.g. appropriate planning to be physically active [26]; suitable goal setting; and commitment to being physically active [55]. These women would also benefit from exploring the reasons, goals and outcomes of why they would want to participate in exercise and physical activity (e.g., being thin and toned, physical self-esteem, to lose weight, to be healthy etc.) [56,57].

It could be that the contribution of family responsibilities (e.g. number of children) to total perceived exercise barrier may not only be a function of limited time due to childcare duties, but may also interact with other factors. Key mediating factors may include: employment or career commitments; level of spouse/ partner support in the home context; local provision of accessible PA opportunities; and possibly most importantly, available and affordable childcare. Thus, the relationship between motherhood and perceived barriers to exercise may well be complex, mediated by the mother’s cultural and socioeconomic status [58]. A mother with three children who does not work and can easily afford convenient childcare with her own transport, might be less likely to perceive motherhood as a ‘less-conducive’ environmental barrier to exercise compared to her less privileged counterpart. If being able to afford convenient childcare and having easy access to attractive PA opportunities are associated with socioeconomic and cultural background, then this could explain King *et al.*’s [27] findings in the USA: that care-giving duties correlated with less activity only in the African American sub-group of the 2,912 middle age and older women they studied. Such findings also resonate with the longstanding social class disparity observed in obesity trends for women in the UK, where the social class influence on obesity showed a large gap, with 10% prevalence in Social Class 1 (professional occupations) and around 25% in Class V (routine occupations) [31].

### Limitations

The study has limitations. Findings of cross sectional studies are associations and do not infer causality. The sample size was 100 women selected at a shopping mall in a London suburb. Hence caution needs to be exercised when attempting generalisations due to the extent to representativeness of this sample to the women population in London or the UK. It was essential to minimise respondent burden, so by keeping the questionnaire short, no data was collected on women’s ethnicity, wider range of other socioeconomic data, or other possibly confounding variables. In addition, the EBBS has few items that directly/indirectly consider ‘family responsibilities’ (not necessarily the same as childcare duties). E.g. some women might have no children but have considerable family responsibilities in relation to caring for elderly relatives etc. While other items of the EBBS could imply ‘childcare duties’, the term is not explicitly stated. Further research would need to refine/expand the items to enable the differentiation in a more precise manner between different types/aspects of ‘family’ duties and responsibilities.

## 5. Conclusions

Older adult non-exercising women have significantly higher perceived barriers to exercise than their younger adult counterparts. Hence more sensitive methods of grouping adult women are required in order to achieve meaningful data that accurately reflect different groups’ barriers to exercise within this broad age category. Secondly, interventions to increase women’s PA must also acknowledge that the 20–35 years age groups is not homogenous and that specific interventions may well be needed for each subgroup for a successful increase in PA.

Further, motherhood appears to present a meaningful challenge to maintaining PA. The mechanism/s by which motherhood and family responsibilities impact upon PA behaviour may well be multifaceted and possibly mediated by numerous factors, potentially including women’s socioeconomic and cultural aspects. A more complete understanding of how motherhood contributes to perceived barriers to exercise is required; examining how this relationship is mediated in order to enhance the evidence base for policy makers to effectively target exercise barriers for adult women.

## Figures and Tables

**Table 1.** Demographic characteristics of non-exercising women.

**Characteristic**	**Sample**	
	**Younger adults (20–27 years)**	**Older adults (28–35 years)**
	n = 50	n = 50
Mean age (SD) in Years	23.4 (2.28)	31.8 (2.38)
Mean number of children (SD)	0.4 (0.61)	1.5 (1.07)
Smokers (%)	44%	36%
Educational background		
Secondary education	62%	78%
Tertiary education	38%	22%
Socio-economic class		
Unemployed	24%	24%
Semi-skilled	18%	16%
Skilled	8%	10%
Clerical	24%	26%
Professional	26%	24%

**Table 2.** Perceived Exercise Barrier Scores of Non-Exercising Younger and Older Adult Women.

**Barrier**	Sample*		***p* value**
	**Younger adults (20–27 years)**	**Older adults (28–35 years)**	
Total Barrier Score	1.81 (0.50)	2.06 (0.47)	< 0.05
Exercise milieu	1.50 (0.47)	1.71 (053)	< 0.05
Time expenditure	1.93 (0.68)	2.29 (0.55)	< 0.01
Physical exertion	2.05 (0.64)	2.13 (0.63)	NS
Family discouragement	1.75 (0.73)	2.08 (0.80)	< 0.05

Cells depict Mean (Standard Deviation); NS: not significant; higher scores represent more perception of barrier/s and vice-versa.
